# 2-Hydroxy-4-(Methylthio) Butanoic Acid Isopropyl Ester Supplementation Altered Ruminal and Cecal Bacterial Composition and Improved Growth Performance of Finishing Beef Cattle

**DOI:** 10.3389/fnut.2022.833881

**Published:** 2022-05-04

**Authors:** Xiaoli Qin, Depeng Zhang, Xinjun Qiu, Kai Zhao, Siyu Zhang, Chunlan Liu, Lianqiang Lu, Yafang Cui, Changxiao Shi, Zhiming Chen, Rikang Hao, Yingqi Li, Shunran Yang, Lina Wang, Huili Wang, Binghai Cao, Huawei Su

**Affiliations:** ^1^State Key Laboratory of Animal Nutrition, College of Animal Science and Technology, China Agricultural University, Beijing, China; ^2^Faculty of Engineering and Applied Science, University of Regina, Regina, SK, Canada

**Keywords:** HMBi, steer, feed efficiency, plasma biochemical indexes, rumen fermentation, cecum fermentation, growth performance

## Abstract

The objective of this study was to evaluate the effects of isopropyl ester of 2-hydroxy-4-(methylthio)-butyrate acid (HMBi) on ruminal and cecal fermentation, microbial composition, nutrient digestibility, plasma biochemical parameters, and growth performance in finishing beef cattle. The experiment was conducted for 120 days by a complete randomized block design. Sixty 24-month-old Angus steers (723.9 ± 11.6 kg) were randomly assigned to one of the flowing three treatments: basal diet (the concentrate: 7.6 kg/head·d^−1^, the rice straw: *ad libitum*) supplemented with 0 g/d MetaSmart^®^ (H_0_), a basal diet supplemented with 15 g/d of MetaSmart^®^ (H_15_), and a basal diet supplemented with 30 g/d of MetaSmart^®^ (H_30_). Results showed that the average daily gain (ADG) increased linearly (*P* = 0.004) and the feed conversion ratio (FCR) decreased linearly (*P* < 0.01) with the increasing HMBi supplementation. Blood urea nitrogen (BUN) concentration significantly decreased in the H_30_ group (*P* < 0.05) compared with H_0_ or H_15_. The ruminal pH value tended to increase linearly (*P* = 0.086) on day 56 with the increased HMBi supplementation. The concentrations of ammonia–nitrogen (NH_3_-N), propionate, isobutyrate, butyrate, isovalerate, valerate, and total volatile fatty acid (VFA) were linearly decreased in the cecum (*P* < 0.05). The results of Phylogenetic Investigation of Communities by Reconstruction of Unobserved States (PICRUSt) showed that the abundance of most pathways with a significant difference was higher in the rumen and lower in the cecum in the H_30_ group compared to the H_0_ group, and those pathways were mainly related to the metabolism of amino acids, carbohydrates, and lipids. Correlation analysis showed that ADG was positively associated with the ratio of firmicutes/bacteroidetes both in the rumen and cecum. Additionally, the abundance of *Lachnospiraceae, Saccharofermentans, Lachnospiraceae_XPB1014_group*, and *Ruminococcus_1* was positively correlated with ADG and negatively correlated with FCR and BUN in the rumen. In the cecum, ADG was positively correlated with the abundances of *Peptostreptococcaceae, Romboutsia, Ruminococcaceae_UCG-013*, and *Paeniclostridium*, and negatively correlated with the abundances of *Bacteroidaceae* and *Bacteroides*. Overall, these results indicated that dietary supplementation of HMBi can improve the growth performance and the feed efficiency of finishing beef cattle by potentially changing bacterial community and fermentation patterns of rumen and cecum.

## Introduction

Methionine (Met) has been proved to be an important nutrient for ruminants, which can improve production performance (1, 2). Free Met can be rapidly and completely degraded by complex rumen microorganisms (3). Rumen-protected Met (RPM) products have been used primarily to resist the microbial degradation and increase the amount of available Met for absorption (4).

The isopropyl ester, HMBi, of 2-hydroxy-4-(methylthio)-butyrate acid (HMTBa) is an effective form of Met analog which can supply metabolizable Met for ruminants. About 50% of HMBi is directly absorbed by the rumen wall into the bloodstream as a form of HMTBa, which is converted to methionine in the liver to provide the body with Met (5, 6), and the remaining 50% is hydrolyzed to HMB and utilized by the rumen microorganisms to facilitate the synthesis of microbial protein through altering the abundance of cellulolytic bacteria and the noncellulolytic species in the rumen of dairy cows (7–9). Although the mode of HMBi metabolism in the rumen has been extensively studied, the underlying mechanism of the effect of HMBi on rumen metabolism remains unclear to date.

The studies of HMBi supplementation have been mostly focused on dairy cows, and the benefits of increased milk yield (10), milk protein content and yield (11), and efficiency of nitrogen utilization have been observed (12). However, studies of HMBi or HMB supplementation on beef cattle are scarce, with only a few studies investigating the growth performance in calves and young cattle (13–15). Han et al. (15) reported that HMBi administration improved the growth performance of growing steers.

Very few studies have evaluated the effects of HMBi on beef cattle during the finishing period, especially for those with bodyweight (BW) heavier than 700 kg. In addition, the effect of HMBi on the bacteria of the hindgut was not investigated in the existing studies. The gastrointestinal microorganism is critical for nutrient digestion and absorption in both rumen and hindgut. The cecum could provide up to 8.6% of metabolizable energy intake as an extra energy source for the host's metabolism (16). Hence, the evaluation of both cecal and ruminal bacteria may enable a better understanding of the effects of HMBi on the production performance of beef cattle.

Therefore, the aim of this study was to examine the effects of HMBi supplementation on growth performance, rumen/cecum fermentation, and plasma biochemical indices of finishing beef cattle. We hypothesized that HMBi supplementation would improve the production performance of finishing beef cattle by promoting the metabolism of rumen/cecum and increasing nitrogen deposition.

## Materials and Methods

### Animals, Experimental Design, and Diets

Sixty 24-month-old Angus steers were used in a 120-day experiment according to a randomized complete block design. The steers were blocked by body weight (723.9 ± 11.6 kg) into 15 pens with 4 steers in each pen. Cattle within a pen were randomly allocated to one of the following experimental diets: basal diet without MetaSmart^®^ (H_0_), basal diet + 15 g/steer per day of MetaSmart^®^ (H_15_), basal diet +30 g/steer per day of MetaSmart^®^ (H_30_). MetaSmart^®^ (Adisseo France SAS) was supplied as a dry powder consisting of 57% HMBi and mixed with the concentrate which was fed at 7.6 kg/head·d^−1^ DM. The ingredients and compositions of the basal diet are shown in Table 1. The steers were allowed free access to rice straw and clean water and provided with their experimental rations twice daily at 06:30 and 15:30. Amino acid balance was predicted by the AMTS.Cattle.Professional ration formulation model (AMTS, LLC. Groton, NY) based on the Cornell Net Carbohydrate and Protein System (CNCPS).

**Table 1:** Ingredients and compositions of the basal diet.

**Item^a^**	**Concentrate**	**Rice straw**
**Ingredients, % of DM**
Wheat bran	30.0	
Corn	27.0	
Wheat	22.0	
Barley	17.0	
Fried soybean	3.00	
NaCl	0.40	
Attractant	0.20	
3% Monensin	0.40	
**Composition, % of DM**
CP	13.9	4.10
EE	4.16	1.61
NDF	20.5	68.5
ADF	6.31	44.2
Ash	4.40	14.6
ME, Mcal/kg	2.95	1.98
**AA Composition, % of total AA**
Aspartic acid (Asp)	7.53	10.8
Threonine (Thr)	3.41	5.05
Serine (Ser)	4.23	4.93
Glutamic acid (Glu)	25.9	16.1
Proline (Pro)	7.45	4.94
Glycine (Gly)	4.78	5.73
Alanine (Ala)	5.49	7.10
Cysteine (Cys)	1.00	0.42
Valine (Val)	5.36	6.58
Methionine (Met)	1.50	0.76
Isoleucine (Ile)	3.91	4.86
Leucine (Leu)	8.22	8.32
Tyrosine (Tyr)	2.92	3.63
Phenylalanine (Phe)	5.03	6.04
Lysine (Lys)	3.97	5.85
Histidine (His)	2.61	1.84
Arginine (Arg)	6.73	7.11

a *DM, dry matter; EE, ether extract; CP, crude protein; NDF, neutral detergent fiber; ADF, acid detergent fiber; ME, metabolizable energy; AA, amino acids*.

### Sampling and Analysis

Feed intake of each pen (offered and refused) was monitored daily during the entire experiment. Feed samples were collected once a week. Samples were dried for 48 h at 65°C in a forced-air oven and ground through a 1-mm sieve for later analysis. The dry matter (DM), crude protein (CP), ether extract (EE), ash and acid-insoluble ash (AIA) were determined following the procedure of AOAC (2005), and the neutral detergent fiber (NDF) and acid detergent fiber (ADF) were analyzed using an Ankom A200 fiber analyzer (Ankom Technology Corp., Macedon, NY), using amylase and sodium sulfide in the NDF analysis. The steers were weighed on days 0, 56, and 105 before morning feeding. Feed conversion ratio (FCR) was the ratio of dry matter intake (DMI) to average daily gain (ADG).

Fecal (about 500 g) samples were collected on days 54, 55, and 56 and on days 103, 104, and 105 and then mixed (equal weight basis) by a steer, respectively. About 300 g of fecal sample was dried at 65°C for 48 h, ground to pass a 1-mm screen, and then analyzed for DM, CP, NDF, ADF, ash, and AIA as described above. Total-tract apparent digestibility was calculated based on AIA concentration in diets and feces (17).

Blood samples were collected from a caudal vein on day 56 and day 105 before the morning feeding. Blood samples (about 10 ml) were collected into a heparinized tube each time and then centrifuged at 3,000 × g for 20 min and stored at −80°C for later analysis. Plasma biochemical parameters were determined by an automatic biochemical analyzer (Hitachi 7020; Hitachi Co., Japan) using corresponding detection kits purchased from Beijing Strong Biotechnologies, Inc.

Ruminal contents were collected *via* esophageal tubing 3 h after morning feeding on days 56 and 105. Samples were filtered through 4 layers of cheesecloth and analyzed for pH (Testo 205, Testo AG, Schwarzwald, Germany). The filtrate was divided into three aliquots, which were immediately frozen using liquid nitrogen and then stored at −80°C for DNA extraction and later analysis of ammonia–nitrogen (NH_3_-N) and volatile fatty acids (VFA). VFA were separated and quantified by gas chromatography (GC-2014 Shimadzu Corporation, Kyoto, Japan) using an HP- INNO wax (30.0 m ×320 μm × 0.5 μm, Catalog No: 19091 N-213, Agilent, US) column. NH_3_-N was determined by the method described by Weatherburn (18).

Cecal contents were collected immediately after the slaughter on day 106. The pH value was determined immediately. Samples were divided into three portions, snap-frozen in liquid nitrogen, and then stored at −80°C until DNA extraction and for later analysis of NH_3_-N and VFA.

### DNA Extraction and 16s rRNA Pyrosequencing

A total of 1 ml sample of rumen fluid and cecum content were centrifuged at 1,000 × g for 10 min to discard sediment. After removing the sediment, the clear supernatant extract was removed by centrifugation at 12,000 × g for 10 min. Subsequently, the DNA of homogenized rumen fluid was extracted using PowerSoil DNA Isolation Kit (MoBio Laboratories, Carlsbad, CA) according to the manufacturer's protocol. The purity and quality of the genomic DNA were checked on 1% agarose gels and a NanoDrop spectrophotometer (Thermo Scientific). Bacterial 16S rRNA genes of the V3–V4 region were amplified from extracted DNA using the 338F primers (5′-ACTCCTACGGGAGGCAGCAG−3′) and 806R primers (5′-GGACTACHVGGGTWTCTAAT-3′). For each sample, an 8-digit barcode sequence was added to the 5'-end of the forward and reverse primers (provided by Allwegene Company, Beijing). PCR reactions were performed in triplicate with 25 μl mixture containing 12.5 μl 2 × Taq PCR MasterMix, 3 μl BSA (2 ng/μl), 1 μl forward primer (5 μ*M*), 1 μl reverse primer (5 μ*M*), 2 μl template DNA, and 5.5 μl ddH_2_O. Cycling parameters were 95°C for 5 min, followed by 28 cycles of 95°C for 45 s, 55°C for 50 s and 72°C for 45 s with a final extension at 72°C for 10 min. Then, the PCR products were checked for size and specificity by agarose gel electrophoresis and purified using an Agencourt AMPure XP Kit. Finally, deep sequencing was performed on the Miseq platform at Allwegene Company (Beijing). After the run, image analysis, base calling, and error estimation were performed using Illumina Analysis Pipeline Version 2.6.

### Data Analysis

Raw data were first screened and the sequences were removed from consideration if they were shorter than 120 bp with a low quality score ( ≤ 20), containing ambiguous bases or mismatching to primer sequences and barcode tags, and separated through the sample-specific barcode sequences. Then the data were merged with the minimum overlap that was set to 10 bp, and the mismatch rate was 0.1 by Pear (v0.9.6) software. Chimeric reads were identified by the UCHIME algorithm of Vsearch (v2.7.1) software and removed. Qualified reads were clustered into operational taxonomic units (OTUs) at a similarity level of 97% (19) using the Uparse algorithm of Vsearch (v2.7.1) software. The Ribosomal Database Project (RDP) Classifier tool was used to classify all OTUs into different taxonomic groups against the SILVA128 database.

QIIME (v1.8.0) was used to generate rarefaction curves and to calculate the richness and diversity indices based on the OTU information. Based on the results of taxonomic annotation and relative abundance, R (v3.6.0) software was used for bar-plot diagram analysis. To examine the similarity between different samples, clustering analysis and principal coordinates analysis (PCoA) were analyzed by R (v3.6.0) based on the OTU information from each sample.

Phyton (V2.7) software was used for Linear Discriminant Analysis Effect Size (LEfSe) analysis, and a threshold of 3.0 was used to determine the significant bacterial taxa. To gain a more fundamental understanding of the bacterial microbiota, we predicted the function of bacteria based on the Kyoto Encyclopedia of Genes and Genomes (KEGG) database using Phylogenetic Investigation of Communities by Reconstruction of Unobserved States (PICRUSt2). Differential abundance of KEGG pathway was tested by Wilcoxon rank and a *p*-value <0.05 was considered statistically significant. Spearman Correlations between growth performance and major bacteria were tested with the cor function in the psych package of R (v.3.6.0), and the correlation heatmap was drawn by R software ggplot2 package. Significant correlations were identified by Spearman's test *(P* < 0.05).

### Statistical Analysis

Feed intake, nutrient digestibility, ruminal and cecal fermentation, production performance, and plasma biochemical index data were analyzed by using PROC MIXED of SAS (version 9.1; SAS Institute Inc., Cary, NC) for variance analysis with the day as the repeated measure, assuming an AR (1) covariance structure. The following model was used:


(1) $$\begin{array}{r} Yijk=\mu +B_{\text{i}}+\tau_{\text{j}}+B_{\text{τ}ij}+W_{\text{k}}+\tau W_{\text{jk}}+e_{\text{ijk}} \end{array}$$

where Y_ijk_ is the dependent variable, μ is the overall mean, Bi is the block, τ_j_ is the jth treatment (diet), B_τij_ is the block × treatment interaction, W_k_ is the sampling week, τW_jk_ is the treatment × week interaction, and e_ijk_ is the random the error term assumed to be normally distributed. Block and block × treatment effects were random, whereas all others were fixed. All data are presented as least squares means. Significant differences among treatments were declared at *p* ≤ 0.05. Differences at 0.05 < *P* ≤ 0.10 were considered a trend toward significance. When the main effect of treatment was significant, means separation tests were conducted using the PDIFF procedure of SAS. After this, mixed linear models (PROC MIXED procedures) were used for analysis, followed by orthogonal comparisons. Orthogonal contrasts were used to partition the main effect of HMBi amount into linear or quadratic effects.

## Results

### Predicted Duodenal Flows of Amino Acids

According to the AMTS.Cattle.Professional ration formulation model based on the CNCPS, the small intestinal essential amino acid flows and proportions of metabolizable protein were predicted for the three treatment diets in Table 2. The ratio of Lysine (Lys) to Met and the Met flow were 2.94:1 and 27.9 g/d, respectively, and the proportion of Met in metabolizable protein (MP) was 3.31% in H_0_. The ratio of Lys to Met and the Met flow were 2.53:1 and 34.6 g/d, respectively, and the proportion of Met in MP was 4.09% in H_15_. The ratio of Lys to Met and the Met flow were 2.21:1 and 41.2 g/d, respectively, and the proportion of Met in MP was 4.85% in H_30_.

**Table 2:** Duodenal flows of digestible EAA as predicted by AMTS. Cattle. Professional software.

**AA^b^**	**Diet^a^**					
	**H**_**0**_		**H**_**15**_		**H**_**30**_	
	**AA flow**,	**MP^c^**,	**AA flow**,	**MP**,	**AA flow**,	**MP**,
	**g/d**	**%**	**g/d**	**%**	**g/d**	**%**
Met	27.90	3.31	34.60	4.09	41.20	4.85
Lys	77.50	9.19	77.50	9.15	77.40	9.11
Arg	68.90	8.17	68.90	8.14	68.80	8.10
His	29.40	3.48	29.30	3.46	29.30	3.45
Ile	57.80	6.85	57.80	6.83	57.70	6.79
Leu	84.40	10.0	84.40	9.97	84.30	9.92
Phe	56.40	6.68	56.40	6.66	56.30	6.63
Thr	53.90	6.39	53.90	6.37	53.90	6.34
Trp	18.40	2.18	18.40	2.17	18.40	2.17
Val	65.20	7.73	65.20	7.70	65.10	7.66
Lys: Met		2.94:1		2.53:1		2.21:1

a *Treatments were: H_0_, basal diet without MetaSmart^®^ H_15_, basal diet supplemented with 15 g/d MetaSmart^®^ H_30_, basal diet supplemented with 30 g/d MetaSmart^®^*.

b *AA, amino acid. Met, Methionine; Lys, Lysine; Arg, Arginine; His, Histidine; Ile, Isoleucine; Leu, Leucine; Phe, Phenylalanine; Thr, Threonine; Trp, Tryptophan; Val, Valine*.

c *MP, metabolizable protein*.

### Growth Performance and Feed Efficiency

The growth performance and feed intake data are presented in Table 3. The steers were provided *ad libitum* access to rice straw after the concentrate was fully consumed (7.6 kg/head·d^−1^) so that the change in DMI was due to the difference in rice straw intake. However, no significant differences were detected among groups in terms of DMI during the entire trial period. With the increasing supplementation of HMBi, the final BW and ADG were significantly increased with the decrease in FCR from day 0 to day 105 (linear, *p* < 0.01). Additionally, the positive effects were particularly significant in the latter stage of the experiment (day 56 to day 105).

**Table 3:** Effect of supplemental HMBi on the growing performance of beef cattle.

**Item**	**Treatment^1^**			**SEM^2^**	**P-value^3^**	
	**H_**0**_**	**H_**15**_**	**H_**30**_**		**Linear**	**Quadratic**
**Body weight, kg**						
Initial	724.20	724.25	723.25	0.39	0.122	0.302
d 56	748.90	749.10	750.75	3.06	0.681	0.852
d 105	768.25^b^	778.30^ab^	781.30^a^	3.14	0.019	0.386
**Dry matter intake (DMI), kg/d**						
d 0- d56	9.87	9.85	0.09	0.16	0.371	0.509
d 56-d 105	10.89	0.98	1.01	0.12	0.518	0.877
d 0-d 105	10.35	0.37	0.52	0.13	0.372	0.713
**Average daily gain (ADG), kg/d**						
d 0- d56	0.44	0.44	0.49	0.05	0.500	0.726
d 56-d 105	0.40^b^	0.60^a^	0.62^a^	0.04	0.003	0.117
d 0-d 105	0.42^b^	0.52^a^	0.55^a^	0.03	0.004	0.387
**Feed conversion ratio (DMI/ADG)**						
d 0- d56	4.03	24.41	20.83	3.13	0.484	0.614
d 56-d 105	8.80^a^	19.04^ab^	17.89^b^	4.01	<0.001	0.734
d 0-d 105	5.10^a^	0.41^ab^	19.13^b^	1.31	0.007	0.308

1 *Treatments were: H0, basal diet without MetaSmart^®^ H15, basal diet supplemented with 15 g/d MetaSmart^®^ H30, basal diet supplemented with 30 g/d MetaSmart^®^*.

2 *SEM, standard error of the mean*.

3 *Significant at P ≤ 0.05*.

a, b *Means within a row followed by different lower-case letters differ significantly from each other (P < 0.05)*.

### Nutrient Apparent Digestibility

The total-tract apparent digestibility is presented in Table 4. There were no significant differences among treatments (*p* > 0.10). However, the digestibility of DM and CP showed a numerically linear increase with the increase of HMBi supplementation in animals.

**Table 4:** Effects of HMBi supplementation on total tract apparent digestibility (%) of beef cattle.

**Item**	**Treatment^a^, ^d^**			**SEM^b^**	***P*** **-value^c^**	
	**H_**0**_**	**H_**15**_**	**H_**30**_**		**Linear**	**Quadratic**
DM	59.10	61.37	61.90	1.63	0.249	0.672
EE	62.34	61.83	63.18	2.10	0.784	0.724
CP	60.60	62.90	64.63	1.85	0.150	0.905
NDF	56.59	56.75	55.67	2.04	0.754	0.808
ADF	51.04	51.24	49.18	1.94	0.510	0.644

a *Treatments were: H_0_, basal diet without MetaSmart^®^ H_15_, basal diet supplemented with 15 g/d HMBi; H_30_, basal diet supplemented with 30 g/d MetaSmart^®^*.

b *SEM, standard error of the mean*.

c *Significant at P ≤ 0.05*.

d *DM, dry matter; EE, ether extract; CP, crude protein; NDF, neutral detergent fiber; ADF, acid detergent fiber*.

### Fermentation in Rumen and Cecum

Most of the parameters in rumen fermentation in this experiment were not affected by diet, except that the rumen pH value tended to increase linearly (*p* = 0.086) on day 56 with the increase of HMBi supplementation (Table 5). In cecum, the concentrations of NH_3_-N, propionate, isobutyrate, butyrate, isovalerate, valerate, and total VFA were significantly decreased with the increase of HMBi supplementation (linear, *p* < 0.05). The ratio of acetate to propionate tended to decrease linearly in the above condition (*p* = 0.056).

**Table 5:** Effect of supplemental HMBi on ruminal and cecal fermentation of beef cattle.

**Item**	**Treatment^1^**			**SEM^2^**	***P*** **-value^3^**	
	**H_**0**_**	**H_**15**_**	**H_**30**_**		**Linear**	**Quadratic**
**Rumen**						
pH (56 d)	5.84	6.05	6.09	0.09	0.086	0.484
pH (105 d)	6.09	6.14	6.22	0.09	0.309	0.896
NH_3_-N,^5^ mg/dL	10.13	11.58	9.61	0.97	0.711	0.175
Acetate	60.66	55.85	61.72	3.62	0.839	0.252
Propionate	19.39	18.80	20.60	1.43	0.559	0.507
Butyrate	10.06	9.44	10.46	0.56	0.623	0.258
Valerate	1.12	1.09	1.18	0.11	0.700	0.647
Isovalerate	1.21	1.19	1.25	0.11	0.822	0.741
Total VFA	95.55	89.28	98.20	5.59	0.743	0.289
Acetate/propionate	3.11	2.91	3.00	0.10	0.444	0.275
**Cecum**						
pH	6.92	6.97	6.88	0.41	0.548	0.157
NH_3_-N,^5^ mg/dL	2.13^a^	1.84^ab^	1.68^b^	0.14	0.028	0.696
Acetate	32.98	30.18	26.77	0.45	0.103	0.912
Propionate	8.92^a^	7.78^ab^	6.56^b^	0.12	0.011	0.955
Isobutyrate	0.97^a^	0.91^ab^	0.75^b^	0.01	0.044	0.615
Butyrate	2.35^a^	1.83^ab^	1.54^b^	0.04	0.009	0.650
Isovalerate	1.19^a^	1.07^ab^	0.86^b^	0.02	0.024	0.687
Valerate	0.90^a^	0.81^ab^	0.66^b^	0.01	0.039	0.609
Total VFA	47.31	42.59	37.14	0.63	0.031	0.924
Acetate/propionate	3.73	3.91	4.08	0.12	0.056	0.98

1 *Treatments were: H_0_, basal diet without MetaSmart^®^ H_15_, basal diet supplemented with 15 g/d MetaSmart^®^ H_30_, basal diet supplemented with 30 g/d MetaSmart^®^*.

2 *SEM, standard error of the mean*.

3 *Significant at P ≤ 0.05*.

^4^VFA, volatile fatty acids.

5 *NH_3_-N, Ammonia-nitrogen.*

a, b *Means within a row followed by different lower-case letters differ significantly from each other (P < 0.05)*.

### Plasma Biochemical Index

The plasma biochemical indexes are presented in Table 6. Blood urea nitrogen (BUN) presented a quadratic effect in response to the increasing HMBi supplementation (*P* = 0.047). Compared with the other two groups, BUN concentration was decreased in steers fed 30 g MetaSmart^®^. There was no significant effect of HMBi on the concentration of other plasma biochemical parameters.

**Table 6:** Effects of supplemental HMBi on plasma biochemical indexes of beef cattle.

**Item^4^**	**Treatment^1^**			**SEM^2^**	***P*** **-value^3^**	
	**H_**0**_**	**H_**15**_**	**H_**30**_**		**Linear**	**Quadratic**
ALT, U/L	27.4	29.3	27.5	1.58	0.983	0.351
AST, U/L	72.3	69.1	68.1	2.36	0.237	0.716
TP, umol/L	65.4	66.2	66.3	1.76	0.710	0.858
TC, mmol/L	4.22	4.16	4.19	0.18	0.927	0.830
TG, mmol/L	0.27	0.25	0.26	0.01	0.565	0.191
BUN, mmol/L	4.47^a^	4.81^a^	4.15^b^	0.18	0.238	0.047
HDLC, mmol/L	1.47	1.47	1.45	0.06	0.762	0.907
LDLC, mmol/L	1.18	1.19	1.19	0.05	0.906	0.940
NEFA, mmol/L	0.71	0.72	0.68	0.04	0.661	0.540

1 *Treatments were: H_0_, basal diet without MetaSmart^®^ H_15_, basal diet supplemented with 15 g/d HMBi; H_30_, basal diet supplemented with 30 g/d MetaSmart^®^*.

2 *SEM, standard error of the mean*.

3 *Significant at P ≤ 0.05*.

4 *ALT, alanine aminotransferase; AST, aspartate aminotransferase; TP, total protein; TC, Cholesterol; TG, Triglyceride; BUN, blood urea nitrogen; HDLC, high-density lipoprotein cholesterol; LDLC, low-density lipoprotein; NEFA, non-esterified fatty acids*.

a, b *Means within a row followed by different lower-case letters differ significantly from each other (P < 0.05)*.

### Microbial Diversities of Rumen and Cecum Microbiota

**Figure 1:**
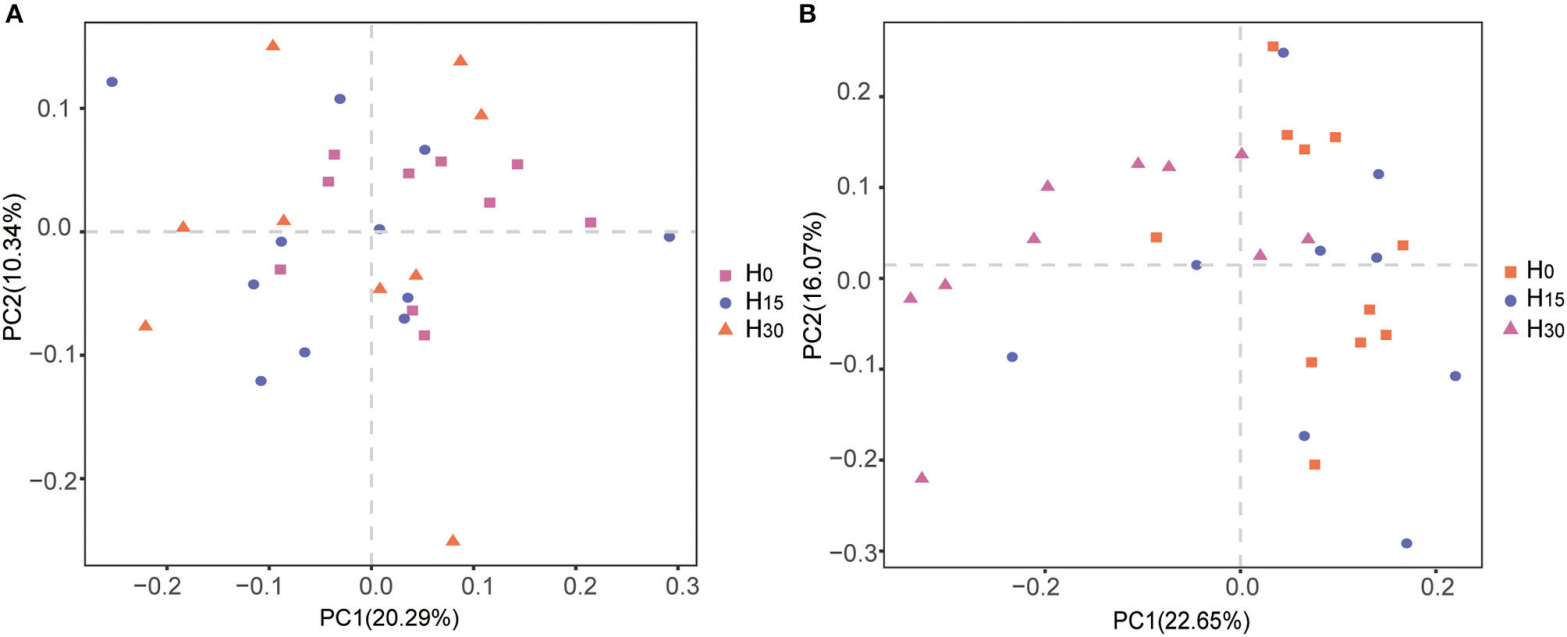
Principal coordinate analysis (PCoA) of the bacterial community structures and relationship in the rumen **(A)** and cecum **(B)**.

In the α diversity index, the addition of HMBi did not affect the microbial diversity indexes of chao1, observed_species, PD_whole_tree, and Shannon, compared with the control group both in rumen and cecum (Table 7). PCoA analysis showed that there were no significant differences in the β-diversity analysis of the microbial community in the rumen (Figure 1A). In cecum, the microbial composition in the H_30_ treatment was distinguished from that in the H_0_ treatment (Figure 1B).

**Table 7:** Effects of supplemental HMBi on the diversity index of bacterial communities in rumen and cecum.

**Item**	**Treatmen^a^**			**SEM^b^**	***P*** **-value^c^**	
	**H_**0**_**	**H_**15**_**	**H_**30**_**		**Linear**	**Quadratic**
**Rumen**
chao1	2,336.83	2,308.84	2,339.48	25.7	0.76	0.15
observed_species	1,815.29	1,800.24	1,824.85	21.1	0.76	0.13
PD_whole_tree	140.08	139.27	139.82	1.32	0.87	0.15
shannon	8.30	8.36	8.37	0.06	0.77	0.12
simpson	0.99	0.99	0.99	0.001	0.62	0.54
**Cecum**
chao1	1,518.94	1,413.35	1,548.07	57.42	0.74	0.14
observed_species	1,204.8	1,109.31	1,268.79	52.28	0.41	0.08
PD_whole_tree	94.16	88.24	96.66	3.34	0.61	0.11
shannon	7.71	7.55	7.96	0.15	0.29	0.18
simpson	0.98	0.98	0.99	0.002	0.22	0.64

a *Treatments were: H_0_, basal diet without MetaSmart^®^ H_15_, basal diet supplemented with 15 g/d HMBi; H_30_, basal diet supplemented with 30 g/d MetaSmart^®^*.

b *SEM, standard error of the mean*.

c *Significant at P ≤ 0.05*.

### Compositions of Rumen and Cecum Microbiota

**Figure 2:**
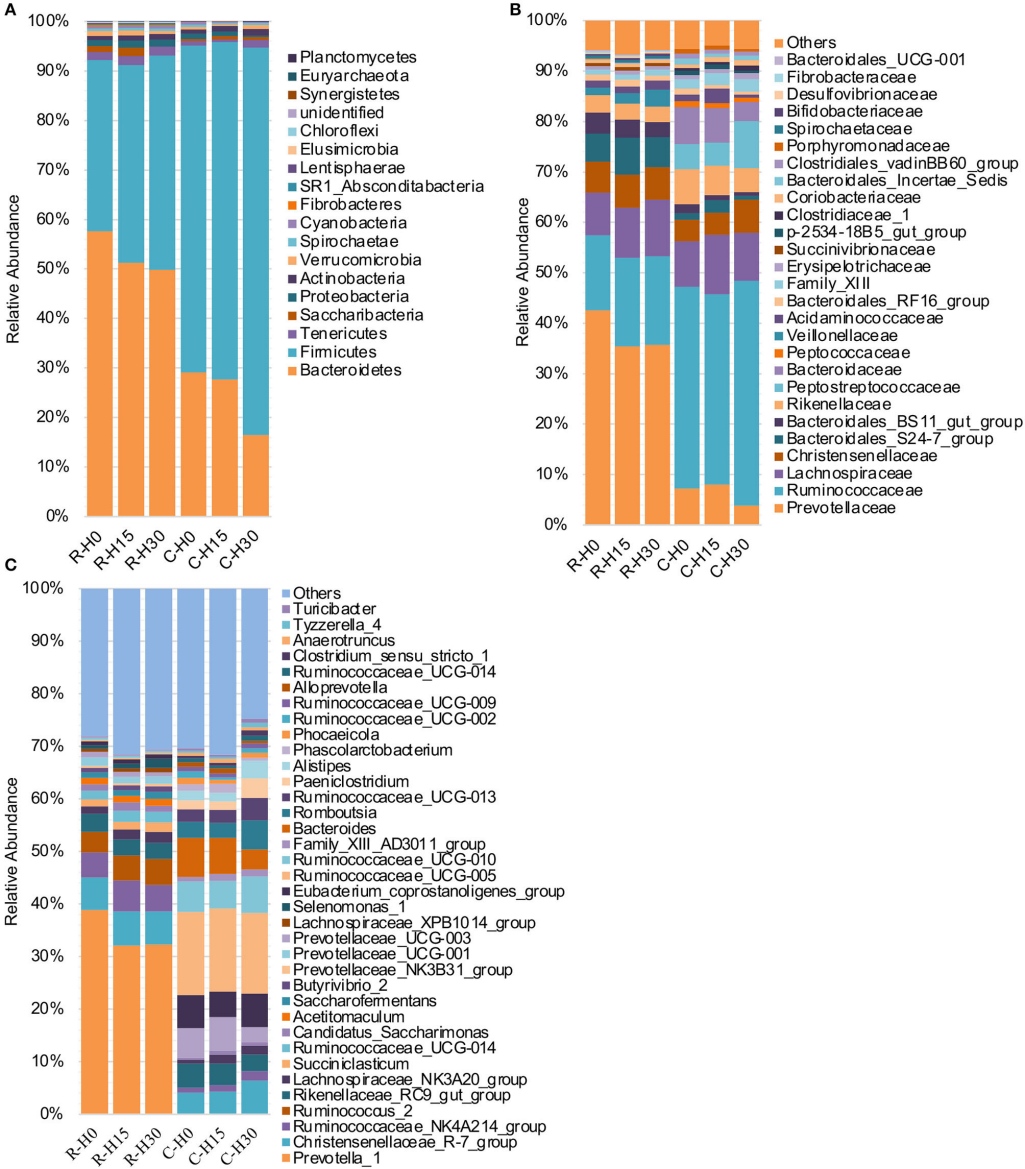
Microbial composition of different groups. Each bar represents the average relative abundance of each bacterial taxon within a group. **(A)** Taxa assignments at the phylum level. **(B)** Taxa assignments at the family level. **(C)** Taxa assignments at the genus level. H0, H15, and H30 indicate basal diet with 0, 15, 30 g/d MetaSmart^®^. R and C indicate rumen and cecum.

**Figure 3:**
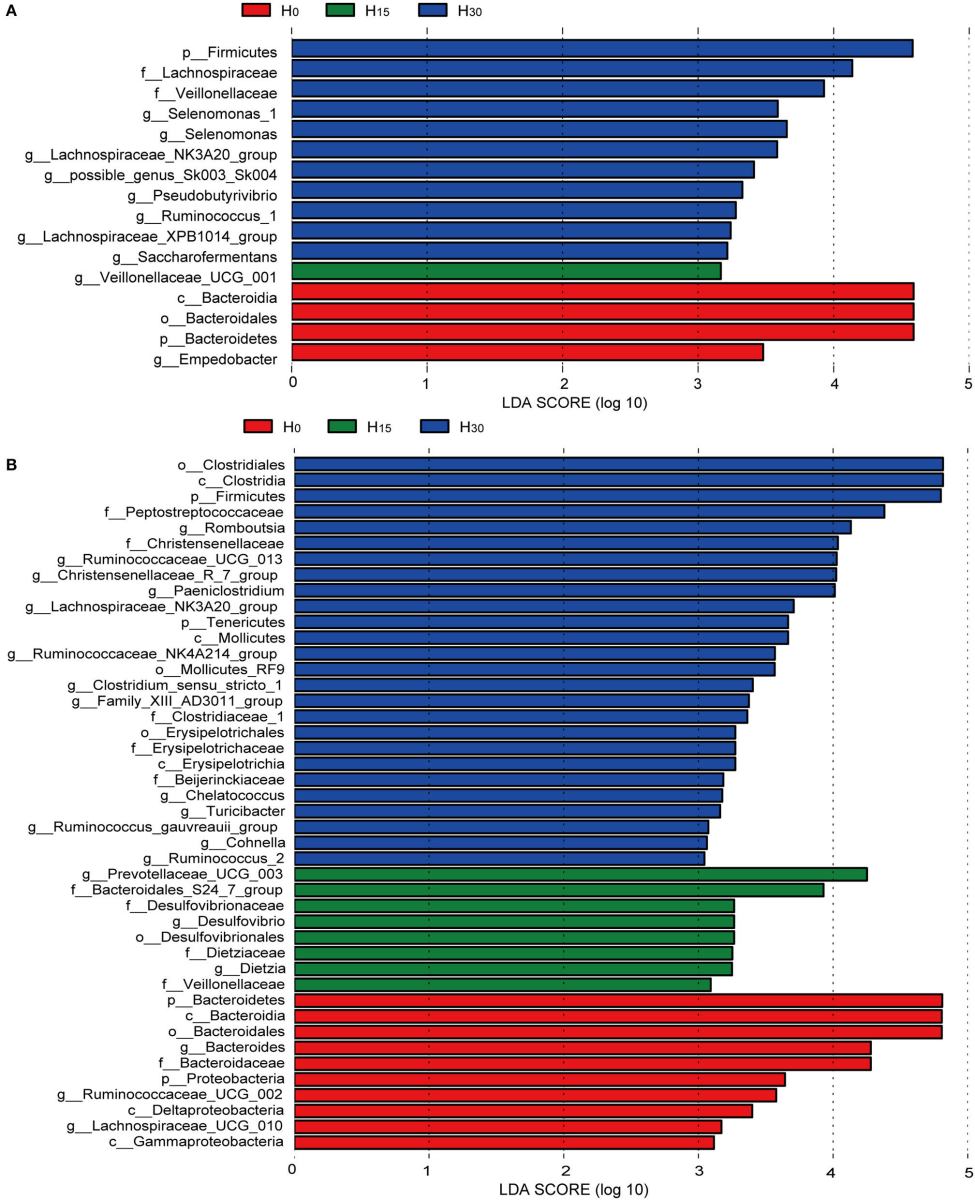
Bacterial taxa significantly differentiated in the rumen **(A)** and cecum **(B)** identified by linear discriminant analysis effect size (LEfSe). Histogram of the LDA scores computed or bacterial taxa differentially abundant among different treatment groups.

The dominant Bacteroidetes and Firmicutes phyla consist of ~90% of the total OTUs both in the rumen and cecum. In terms of the assignment at the phylum level, the increasing HMBi supplementation significantly increased the abundance of the Firmicutes phylum (Linear, *P* = 0.008) and decreased the abundances of Bacteroidetes, Lentisphaerae, and Euryarchaeota (Linear, *P* < 0.05) in the rumen (**Figure 3A**; Supplementary Table 1). In the cecum, the abundances of Bacteroidetes and Proteobacteria were significantly decreased (Linear, *P* < 0.05), while Firmicutes and Actinobacteria were significantly increased (Linear, *P* < 0.01) (Figure 2A; Supplementary Table 1). Additionally, the abundance of Tenericutes had a quadratic relationship with HMBi supplementation (*p* < 0.05).

At the family level (Figure 2B; Supplementary Table 2), the increasing inclusion of HMBi linearly increased the relative abundance of *Lachnospiraceae* (*P* < 0.01), *Veillonellaceae* (*P* = 0.01), and *Bifidobacteriaceae* (*P* = 0.03) in the rumen (Figure 3A), while it linearly decreased the relative abundances of *Bacteroidales_BS11_gut_group* (*P* = 0.04), *Fibrobacteraceae* (*P* = 0.05), and *Bacteroidales_UCG-001*(*P* = 0.02). The relative abundances of *Prevotellaceae* (*P* = 0.08) and *Ruminococcaceae* (*P* = 0.07) had linearly decreasing and increasing trends, respectively. The relative abundance of *Bacteroidales_S24-7_group* had a quadratic relationship with HMBi supplementation (*P* = 0.04). In cecum (Figure 3B), the abundances of *Peptostreptococcaceae, Christensenellaceae*, and *Clostridiaceae_1* were linearly increased (*P* < 0.05), whereas the abundance of *Bacteroidaceae* and *Porphyromonadaceae* were linearly decreased (*P* < 0.05). The abundance of *Bacteroidales_S24-7_group* had a quadratic relationship with the increasing HMBi addition (*P* < 0.01).

At the genus level (Figure 2C; Supplementary Table 3), dietary supplementation of HMBi tended to decrease the abundances of *Prevotella_1* (*P* = 0.09) and decreased *Prevotellaceae_UCG-003* (*P* < 0.01) and increased the abundances of *Lachnospiraceae_NK3A20_group, Saccharofermentans, Lachnospiraceae_XPB1014_group, Pseudobutyrivibrio, and Anaerovibrio* in the rumen (*P* < 0.05, Figure 3A). The relative abundance of *Candidatus_Saccharimonas* had a quadratic relationship with HMBi supplementation in the rumen (*P* < 0.01). In the cecum, dietary supplementation of HMBi linearly reduced the relative abundance of Bacteroides (*P* < 0.05) and increased *Christensenellaceae_R-7_group, Ruminococcaceae_UCG-013, Paeniclostridium, Alistipes, Ruminococcaceae_NK4A214_group, Family_XIII_AD3011_group, Clostridium_sensu_stricto_1, Tyzzerella_4*, and *Turicibacter* (*P* < 0.05, Figure 3B). Moreover, *Eubacterium_coprostanoligenes_group, Romboutsia*, and *Paeniclostridium* had a quadratic relationship with HMBi supplementation (*P* < 0.05).

### Metabolic Functions

**Figure 4:**
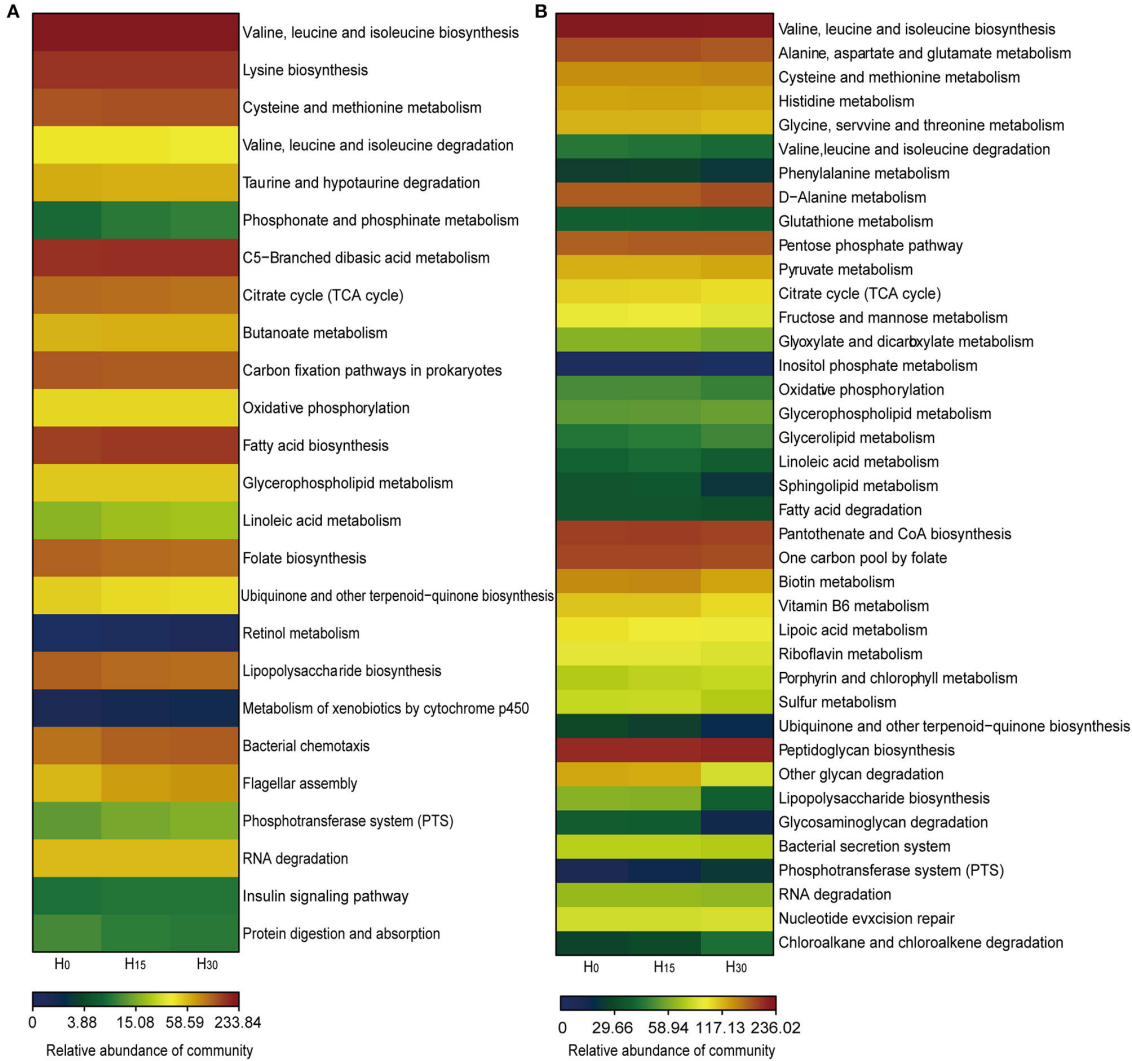
The relative abundance of KEGG pathways of microbiota in the rumen **(A)** and cecum **(B)**. Only the KEGG pathways with a relative abundance above 0.1% and significant differences (*p* < 0.05) are presented.

The tertiary metabolism is shown in Figure 4 and Supplementary Table 4. Compared with the H_0_ group, the H_30_ group had higher abundances of pathways related to valine, leucine, and isoleucine biosynthesis; lysine biosynthesis; cysteine and methionine metabolism; and a lower valine, leucine, and isoleucine degradation in the rumen. Additionally, the abundances of most pathways in the rumen were significantly higher in the H_30_ group than that in the H_0_ group.

In the cecum, the abundance of most pathways increased in the H_15_ group and decreased in the H_30_ group than that of the H_0_ group (Figure 4; Supplementary Table 5). For instance, the H_15_ group had higher abundances of pathways related to valine, leucine, and isoleucine biosynthesis; alanine, aspartate, and glutamate metabolism; cysteine and methionine metabolism; histidine metabolism; phenylalanine metabolism and glutathione metabolism; and lower abundances of glycine, serine, and threonine metabolism and the valine, leucine, and isoleucine degradation. In contrast, all the abundance of pathways mentioned above, except for cysteine and methionine metabolism and d-alanine metabolism, decreased in the H_30_ group than that in the H_0_ group.

### Correlation Analysis

**Figure 5:**
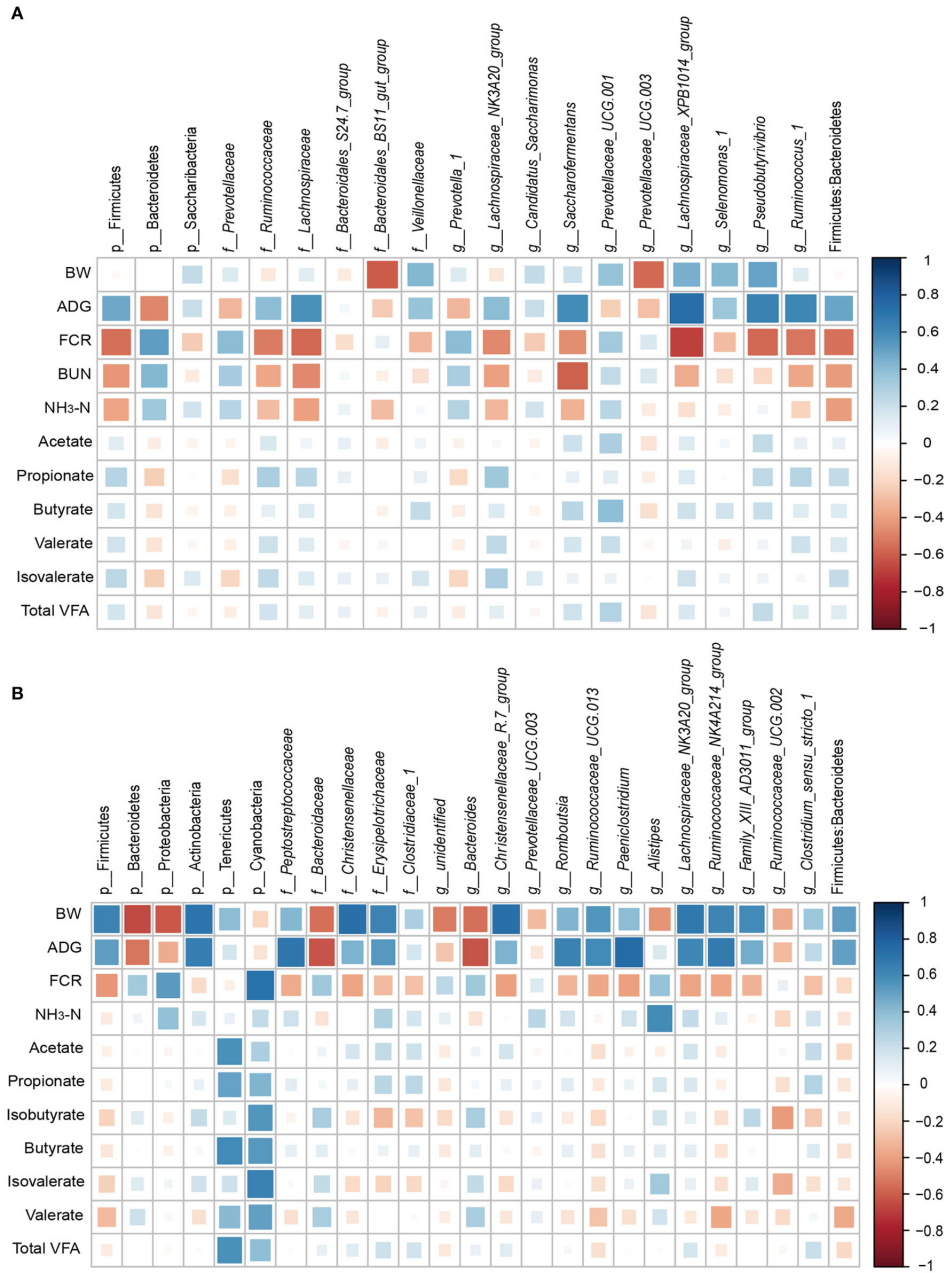
Correlation between physiological parameters/production performance and bacteria abundance in the rumen **(A)** and cecum **(B)**. Only significant correlations and bacteria abundances > 0.5% are shown. Strong correlations are indicated by large squares, weak correlations by small squares. The scale colors denote whether the correlation is positive (closer to 1, blue squares) or negative (closer to −1, red squares) between the bacteria and the efficiency parameters.

The relationships between physiological parameters/growth performance and major bacteria were evaluated (Figure 5). In the rumen, ADG was positively associated with the abundances of family *Lachnospiraceae* (r = 0.58; *P* = 0.02), genus *Saccharofermentans* (r = 0.59; *P* = 0.02), genus *Lachnospiraceae_XPB1014_group* (r = 0.74; *P* < 0.01), genus *Pseudobutyrivibrio* (r = 0.65; *P* < 0.01), and genus *Ruminococcus_1* (r = 0.62; *P* = 0.01). FCR was positively correlated with the abundances of phylum Bacteroidetes (r = 0.52; *P* < 0.01) and negatively with the abundances of phylum Firmicutes (r = −0.54; *P* = 0.04), family *Lachnospiraceae* (r = −0.56; *P* = 0.03), genus *Lachnospiraceae_XPB1014_group* (r = −0.68; *P* < 0.01), genus *Pseudobutyrivibrio* (r = −0.57; *P* = 0.03), genus *Ruminococcus_1* (r = −0.52; *P* = 0.04), and the ratio of Firmicutes: Bacteroidetes (r = −0.53; *P* = 0.04).

In the cecum, the results showed that the concentrations of acetate and butyrate were positively associated with the Tenericutes phylum (r = 0.57; *P* < 0.05). The isovalerate content (r = 0.64, *P* = 0.02) and FCR (r = 0.71; *P* < 0.01) were positively correlated with the abundance of the Cyanobacteria phylum. ADG was positively correlated with the abundance of *Peptostreptococcaceae* (r = 0.69; *P* = 0.01), *Romboutsia* (r = 0.65; *P* = 0.02), *Ruminococcaceae_UCG-013* (r = 0.61; *P* = 0.04), and *Paeniclostridium* (r = 0.74; *P* < 0.01), and negatively with the abundance of *Bacteroidaceae* and *Bacteroides* (r = −0.62; *P* = 0.03). BW was negatively associated with the phyla Bacteroidetes (r = −0.66; *P* = 0.02) and Proteobacteria (r = −0.62; *P* = 0.03), and positively correlated with Actinobacteria, family *Christensenellaceae*, family *Erysipelotrichaceae*, genus *Christensenellaceae_R-7_group*, genus *Ruminococcaceae_UCG-013*, genus *Lachnospiraceae_NK3A20_group*, genus *Ruminococcaceae_NK4A214_group*, genus *Family_XIII_AD3011_group* (r > 0.56; *P* < 0.05).

## Discussion

### Amino Acid Balance and Growth Performance

In conventional diets, Met is considered as the first-limiting AA for milk protein synthesis in dairy cows (20). Diets supplemented with RPM or methionine analogs (i.e., HMBi and HMBa) could improve the production performance. Feeding HMBi increased milk protein percentage and yield (11) by improving N efficiency (12). In this study, the H_30_ group increased ADG by 228 g/day compared with the H_0_ group. Similarly, Han et al. (15) found that the addition of HMBi increased the total gain and the ADG of Holstein growing steers. Chen et al. (21) reported that HMBi supplementation improved the growth performance of goats. In the current study, ADG of Angus steers significantly increased along with the HMBi supplementation, which was consistent with the above reports.

Both the insufficient amount and unbalanced profile of absorbed amino acids in the small intestine were the factors that restrict the protein deposition in muscle tissues (20). According to the AMTS.Cattle.Professional ration formulation model based on the CNCPS, the recommended ratio of Lys and Met was 2.7:1. In the present study, the ratio of Lys: Met was 2.94:1 in the H_0_ group, which indicated that Met may be insufficient. With the increased supplementation of HMBi, the ratio of Lys: Met decreased to 2.53:1 (H_15_) and 2.21:1 (H_30_). Thus, the increase in ADG may be partially attributed to the improved duodenal digestible amino acids profile *via* HMBi supplementation. Met not only serves as a precursor for protein synthesis, but also functions as a signaling factor that can promote nitrogen deposition. Previous research in dairy cows showed that Met improved milk protein synthesis through the mammalian target of rapamycin (mTOR) signaling pathway (22, 23). Moreover, Met is the source of methyl groups (24), which can affect the expression of related genes by providing methyl donors for DNA methylation (25). In the current research, HMBi may promote muscle protein synthesis through related signaling pathways (i.e., mTOR pathway) or its role as a methyl donor.

### Blood Biochemical Index

In this experiment, the BUN concentration was significantly decreased with the increasing HMBi addition, which was consistent with the changes of CP apparent digestibility, total VFA concentration, and ADG. BUN mainly originated from rumen NH_3_-N generated by the degradation of dietary CP and the deamination of AA. The lower BUN concentration in H_30_ indicated that more N was incorporated into metabolizable protein from the rumen microbial protein sources. This is consistent with the results of the correlation analysis that BUN had a significantly negative relationship with *Saccharofermentans*, which increased its abundance in H_30_ compared with H_0_. Moreover, BUN was strongly related to the balance of amino acid absorbed, which could accurately reflect the protein metabolism and nitrogen deposition in the body (26). When the profile of the adsorbed amino acids was unbalanced, the concentration of BUN increased and the utilization efficiency of protein decreased (27). Many studies have found that ruminant diets supplemented with Met decreased the BUN concentration and increased the efficiency of protein utilization (21, 28, 29). Our study was consistent with the research described above, in which HMBi supplementation increased dietary nitrogen utilization efficiency and protein deposition.

### Rumen Fermentation and Bacteria Composition

There is a dependent relationship among dietary composition, microbial richness, and the physiology of the host. Dietary composition is considered an important factor affecting microbial composition and diversity (30). In the present study, the similar bacterial richness and diversity may be attributed to the similar dietary composition.

The impact of HMBi or HMB supplementation on VFA production in the rumen was variable *in vitro* or *in vivo*, in which several studies have shown positive effects (7, 31). Whereas, most studies did not observe any effect of Met analog supplementation on VFA concentrations or profiles, which was consistent with the current study (32, 33). The discrepancy of the effect of HMBi on the ruminal microbes may be explained by the increased bacterial N derived from NH_3_-N, peptides N, and AA but decreased the production of acetate, propionate, and total VFA because a fraction of carbon was being used for AA synthesis (8). Additionally, the produced VFAs had another fate toward *de novo* synthesis of fatty acids (34). The changed structure of the bacterial community may also contribute to the observation that HMBi supplementation did not affect rumen fermentation. In this study, the sum of dominant phyla Bacteroidetes and Firmicutes consist of ~90% of the total OTUs both in the rumen and cecum. An abundance of biosynthesis and membrane transport-related genes were observed in Firmicutes, while Bacteroidetes had numerous carbohydrate-active enzymes used for polysaccharide degradation (35). Parnell and Reimer (36) found that the Bacteroidetes had a negative correlation with the percentage of body fat and body weight. Intriguingly, the H_30_ diet significantly increased the relative abundance of Firmicutes and decreased Bacteroidetes both in the rumen and cecum, thus leading to the increasing ratio of Firmicutes/Bacteroidetes. Especially in cecum, the ratio increased from 2.27 (H_0_) to 4.74 (H_30_). Greater BW and ADG in steers supplemented with HMBi may be partially contributed to the increased ratio of Firmicutes/Bacteroidetes. This was consistent with the previous reports that the ratio of Firmicutes to Bacteroidetes affected energy harvesting in the guts of mice and humans, where a high Firmicutes/Bacteroides ratio was associated with obesity (37, 38).

*Ruminococcus* and *Fibrobacteriaceae* are the major cellulolytic bacteria and play an important role in fiber degradation because they are rich in genes encoding cellulase and hemicellulase. The current study found that the abundance of the *Fibrobacteraceae* family (only accounted for about 0.27%) tended to decrease linearly and the *Ruminococcus* (accounted for 17.51%) tended to increase linearly with the increasing HMBi supplementation, suggesting that the degradation of structural carbohydrates mainly depended on the *Ruminococcus*. The high-starch diet has been recognized to restrict the activity of fiber-degrading bacteria (39, 40). In this research, although the diets contain about 36% starch, the negative impact of high starch content on the activity of cellulolytic bacteria may be partially offset by the positive effects of HMBi. A previous study supported our data that AA might stimulate fibrolytic bacteria (41). Adding Met analog increased the contents of microbial protein and total VFA by potentially stimulating carboxymethylcellulase activity and rumen microbial populations, such as *Fibrobacter succinogenes* and *Ruminococcus flavefaciens*, which belong to *Ruminococcus* and *Fibrobacteriaceae*, respectively (7, 42). The *Ruminococci* was considered the key symbionts of the gut ecosystem (43). *Ruminococcaceae* was more abundant in the cecum and feces from the Angus steers with high feed efficiency (44), which was consistent with our results.

Isobutyrate and isovalerate, known as branched-chain fatty acids (BCFAs), are produced by the deamination of branched-chain amino acids by bacteria and are considered as the indicators of protein fermentation. In cecum, the increase in HMBi supplementation decreased the concentrations of NH_3_-N, propionate, isobutyrate, butyrate, isovalerate, valerate, and total VFA. The possible explanation is that HMBi supplementation promoted the digestion and absorption of the nutrient substrate in the rumen and the small intestine, leading to the decreased amount of the substrates entering the cecum. This was consistent with the results of PICRUSt mentioned above that the H_30_ diet increased the abundances of most pathways in rumen, such as amino acid metabolism, carbohydrate metabolism, and membrane transport, while the H_30_ diet decreased the abundance of most pathways with significant differences in the cecum. Additionally, the decreased total VFA may be partially attributed to the fact that the produced VFAs were rapidly utilized to provide metabolizable energy for the host by simple diffusion or carrier-mediated transport (45, 46). Besides, the altered composition of the bacterial community may also potentially affect VFA production. Correlation analysis presented that Tenericutes and Cyanobacteria were significantly associated with the concentration of acetate, butyrate, and isovalerate.

In summary, HMBi promotes the metabolism and biosynthesis of amino acids, carbohydrates, and lipids in the rumen by potentially stimulating microbial fermentation, such as the increased ratio of Firmicutes/Bacteroidetes and the abundance of *Ruminococcus*, thus leading to greater BW and ADG.

### Correlations Between Growth Performance and Dominant Bacteria

Correlation analysis revealed relationships between fermentation and differential microbial abundances in the rumen and cecum. In this study, rumen microbial abundances had no significant correlation with rumen fermentation. Notably, *Lachnospiraceae, Lachnospiraceae_XPB1014_group, Pseudobutyrivibrio*, and *Ruminococcus_1* were positively correlated with ADG. As discussed above, *Ruminococcus_1* and *Butyrivibrio -Pseudobutyrivibrio* mainly produce acetate by fermenting cellulose and hemicellulose (47, 48). *Lachnospiraceae_XPB1014_Group* was reported to participate in the degradation of fiber (49). Previous research reported that the family *Lachnospiraceae*, including *Lachnospiraceae_ XPB1014_ group*, tended to have greater relative abundance in Nellore steers with low nitrogen retention and in low-feed-efficiency beef cattle (50, 51), which may be explained by the tryptophan metabolism and degradations of valine, leucine, and isoleucine (52). Fowler et al. (8) investigated that HMBi would sustain ruminally available Met and potentially increase the efficiency of microbial protein synthesis. In the current study, the improved ADG and FCR were significantly correlated with the amino acid metabolism pathways, including valine, leucine, and isoleucine biosynthesis pathway, lysine biosynthesis pathway, and cysteine and methionine metabolism pathway, which were more active in the H_30_ group as PICRUSt mentioned above.

In the cecum, the contents of acetate and butyrate were positively associated with Tenericutes, and the isovalerate content and FCR were positively correlated with Cyanobacteria. In this study, the abundances of Tenericutes and Cyanobacteria increased in the H_30_ group. This was inconsistent with the previous research that low abundances of Tenericutes and Cyanobacteria were correlated with high residual feed intake (low feed efficiency) in bulls (53). ADG had a positive relationship with the abundances of family *Peptostreptococcaceae*, including the genera *Rombouts, Paeniclostridium, and Ruminococcaceae_UCG-013*. *Peptostreptococcaceae* was generally considered as the gut symbiotic bacteria because of its role in maintaining the gut homeostasis (54) and it enables to produce a vast amount of ammonia derived from amino acids or peptides (55). This was consistent with the aforementioned results of PICRUSt that the abundance of several typical metabolic pathways in H_15_-fed steers were higher than that of H_30_-fed steers, such as amino acid metabolism, carbohydrate metabolism, and lipid metabolism. These alterations were associated with the improvement of the ADG and FCR. Contrary to our results, Gomes Carvalho Alves et al. (56) reported that *Lachnospiraceae* and *Peptostreptococcaceae*, belonging to *Clostridiale* order, were associated with low efficiency of nitrogen retention in beef cattle. The possible explanation of the inconsistent results was that the supplementation of HMBi compensated the degradation of proteins in the rumen by promoting microbial protein synthesis and stimulated the utilization of absorbed protein for the synthesis of body protein (57). Consistent with the result of ADG, BW had similar trends of correlation with the significantly different bacteria in cecum, such as *Lachnospiraceae_NK3A20_group, Ruminococcaceae_NK4A214_group*, and Bacteroidetes: Firmicutes ratio. Additionally, BW was negatively associated with the abundance of Proteobacteria and positively correlated with Actinobacteria and *Erysipelotrichaceae* family. An increased abundance of Proteobacteria is a potential signature of dysbiosis in gut microbiota (58). The decreased abundance of Proteobacteria with the increased HMBi inclusion indicated that the homeostasis in gut microbiota was improved. *Erysipelotrichaceae* was increased and it was positively correlated with BW. Similarly, Bach et al. (59) observed that *Erysipelotrichaceae* had a positive correlation with feed efficiency.

Taken together, the correlation analysis revealed that the improved production performance is closely related to the impact of HMBi supplementation on the alterations and adjustment of crucial bacterial communities in the rumen and cecum.

## Conclusion

In summary, dietary supplementation of HMBi improved the growth performance and feed efficiency in finishing beef cattle, which can be partially attributed to the altered bacterial community and fermentation patterns of rumen and cecum.

## Data Availability Statement

The metagenomic data are available in the NCBI database under accession PRJNA788170.

## Ethics Statement

The animal protocol was approved by the Animal Care and Use Committee of China Agricultural University (AW81110202-1).

## Author Contributions

XQin, DZ, BC, and HS conceived and designed the experiments. XQin, XQiu, DZ, SZ, CL, LL, YC, CS, ZC, RH, YL, SY, LW, and HW conducted the experiments and performed the statistical analysis of the experimental data. Finally, the paper was written by XQin and was modified by KZ and HS. All authors have read and approved the final manuscript.

## Funding

This work was funded by the National Natural Science Foundation of China (Grant No. 31802084), the National Key R&D Program of China (2018YFD05018000), the Key Technology R&D Program of Ningxia (2017BY078), and China Agriculture Research Systems of MOF and MARA (CARS-37).

## Conflict of Interest

The authors declare that the research was conducted in the absence of any commercial or financial relationships that could be construed as a potential conflict of interest.

## Publisher's Note

All claims expressed in this article are solely those of the authors and do not necessarily represent those of their affiliated organizations, or those of the publisher, the editors and the reviewers. Any product that may be evaluated in this article, or claim that may be made by its manufacturer, is not guaranteed or endorsed by the publisher.
