# Supplementary material for: 2-Hydroxy-4-(Methylthio) Butanoic Acid Isopropyl Ester Supplementation Altered Ruminal and Cecal Bacterial Composition and Improved Growth Performance of Finishing Beef Cattle
Source: Front Nutr. 2022 May 4;9:833881. doi: 10.3389/fnut.2022.833881 (PMC9116427; doi:10.3389/fnut.2022.833881)
Supplement: Supplementary file 1 [file Data_Sheet_1.docx]

Supplementary Material

**Supplementary Table 1.** Effects of supplemental HMBi on bacterial composition in rumen and cecum at phylum level (relative abundance > 0.01%) of beef cattle

| Phylum Name | Treatment^1^ | | | SEM^2^ | *P* - value^3^ | |
| --- | --- | --- | --- | --- | --- | --- |
|  | H_0_ | H_15_ | H_30_ |  | Linear | Quadratic |
| **rumen** |  |  |  |  |  |  |
| Bacteroidetes | 57.59a | 51.29b | 49.21b | 2.00 | 0.01 | 0.41 |
| Firmicutes | 34.56b | 39.86ab | 42.80a | 1.88 | 0.01 | 0.62 |
| Tenericutes | 1.69 | 1.87 | 1.80 | 0.12 | 0.52 | 0.40 |
| Saccharibacteria | 1.21b | 1.67a | 1.18b | 0.12 | 0.84 | 0.01 |
| Proteobacteria | 1.19 | 1.45 | 1.36 | 0.15 | 0.44 | 0.37 |
| Actinobacteria | 0.83 | 1.01 | 1.12 | 0.13 | 0.13 | 0.82 |
| Verrucomicrobia | 0.94 | 0.96 | 0.63 | 0.11 | 0.07 | 0.2 |
| Spirochaetae | 0.57 | 0.5 | 0.51 | 0.07 | 0.54 | 0.65 |
| Cyanobacteria | 0.34 | 0.42 | 0.44 | 0.08 | 0.38 | 0.75 |
| Fibrobacteres | 0.35 | 0.21 | 0.23 | 0.04 | 0.05 | 0.16 |
| SRAbsconditabacteria | 0.2 | 0.28 | 0.29 | 0.06 | 0.32 | 0.59 |
| Lentisphaerae | 0.16a | 0.10b | 0.090b | 0.01 | <0.01 | 0.32 |
| Elusimicrobia | 0.13 | 0.1 | 0.11 | 0.03 | 0.64 | 0.60 |
| Chloroflexi | 0.09 | 0.11 | 0.07 | 0.02 | 0.49 | 0.18 |
| unidentified | 0.07 | 0.08 | 0.09 | 0.02 | 0.22 | 0.82 |
| Synergistetes | 0.04 | 0.04 | 0.05 | 0.01 | 0.31 | 0.89 |
| Euryarchaeota | 0.023a | 0.014b | 0.0072c | 0.000 | <0.01 | 0.84 |
| **cecum** |  |  |  |  |  |  |
| Firmicutes | 66.03bc | 68.11b | 78.22a | 0.01 | 0.01 | 0.28 |
| Bacteroidetes | 29.10a | 27.69a | 16.50b | 0.01 | 0.01 | 0.22 |
| Tenericutes | 0.91ab | 0.54b | 1.51a | 0.01 | 0.03 | 0.01 |
| Actinobacteria | 0.72b | 1.11ab | 1.30a | 0.11 | 0.04 | 0.67 |
| Verrucomicrobia | 0.56 | 0.29 | 0.81 | 0.49 | 0.55 | 0.29 |
| Saccharibacteria | 0.36 | 0.72 | 0.64 | 0.29 | 0.25 | 0.29 |
| Spirochaetae | 0.54 | 0.35 | 0.42 | 0.79 | 0.66 | 0.60 |
| Proteobacteria | 1.20a | 0.90ab | 0.32b | 0.01 | <0.01 | 0.58 |
| Cyanobacteria | 0.52 | 0.18 | 0.19 | 0.10 | 0.06 | 0.26 |
| Chloroflexi | 0.02 | 0.05 | 0.03 | 0.17 | 0.30 | 0.12 |
| Elusimicrobia | 0.02 | 0.01 | 0.02 | 0.39 | 0.31 | 0.35 |
| Synergistetes | 0.003 | 0.012 | 0.003 | 0.13 | 0.87 | 0.05 |
| unidentified | 0.0019 | 0.0013 | 0.0015 | 0.87 | 0.71 | 0.72 |
| Planctomycetes | 0.00026 | 0.00032 | 0.00029 | 0.99 | 0.95 | 0.90 |

^1^ Treatments were: H_0_, basal diet without HMBi; H_15_, basal diet supplemented with 15 g/d HMBi; H_30_, basal diet supplemented with 30 g/d HMBi.

^2^ SEM = standard error of the mean.

^3^ Significant at *P* ≤ 0.05.

**Supplementary Table 2.** Effects of supplemental HMBi on bacterial composition in rumen and cecum at family level (relative abundance > 0.1%) of beef cattle

| Family Name | Treatment^1^ | | | SEM^2^ | *P* - value^3^ | |
| --- | --- | --- | --- | --- | --- | --- |
|  | H_0_ | H_15_ | H_30_ |  | Linear | Quadratic |
| **rumen** |  |  |  |  |  |  |
| *Prevotellaceae* | 42.59 | 35.39 | 35.75 | 2.56 | 0.08 | 0.25 |
| *Ruminococcaceae* | 14.83 | 17.61 | 17.51 | 0.98 | 0.07 | 0.25 |
| *Lachnospiraceae* | 8.42c | 9.89b | 11.26a | 0.44 | <0.01 | 0.93 |
| *Christensenellaceae* | 6.17 | 6.54 | 6.42 | 0.43 | 0.69 | 0.66 |
| *Bacteroidales_S24-7_group* | 5.58b | 7.34a | 5.91b | 0.58 | 0.69 | 0.04 |
| *Bacteroidales_BS11_gut_group* | 4.15a | 3.65ab | 3.03b | 0.34 | 0.04 | 0.90 |
| *Rikenellaceae* | 3.52 | 3.16 | 3.05 | 0.38 | 0.40 | 0.78 |
| *Veillonellaceae* | 1.50bc | 2.02b | 3.36a | 0.32 | <0.01 | 0.32 |
| *Acidaminococcaceae* | 1.37 | 1.36 | 1.85 | 0.24 | 0.18 | 0.41 |
| *Bacteroidales_RF16_group* | 1.13 | 1.31 | 0.99 | 0.19 | 0.61 | 0.32 |
| *Family_XIII* | 1.07 | 1.07 | 1.11 | 0.11 | 0.83 | 0.88 |
| *Erysipelotrichaceae* | 0.67 | 0.78 | 0.70 | 0.14 | 0.87 | 0.59 |
| *Succinivibrionaceae* | 0.59 | 0.70 | 0.65 | 0.12 | 0.72 | 0.60 |
| *Coriobacteriaceae* | 0.60 | 0.66 | 0.64 | 0.08 | 0.76 | 0.70 |
| *Spirochaetaceae* | 0.57 | 0.50 | 0.50 | 0.07 | 0.52 | 0.64 |
| *Bifidobacteriaceae* | 0.22b | 0.35ab | 0.48a | 0.07 | 0.03 | 0.99 |
| *Desulfovibrionaceae* | 0.23 | 0.33 | 0.28 | 0.04 | 0.35 | 0.16 |
| *Fibrobacteraceae* | 0.35 | 0.22 | 0.23 | 0.04 | 0.05 | 0.16 |
| *Bacteroidales_UCG-001* | 0.22a | 0.15b | 0.15b | 0.02 | 0.02 | 0.12 |
| *Bacteroidales_Incertae_Sedis* | 0.13 | 0.11 | 0.13 | 0.02 | 0.81 | 0.42 |
| *Defluviitaleaceae* | 0.10 | 0.14 | 0.11 | 0.02 | 0.77 | 0.22 |
| *Mycoplasmataceae* | 0.09 | 0.13 | 0.12 | 0.02 | 0.43 | 0.43 |
| *Anaeroplasmataceae* | 0.12 | 0.09 | 0.10 | 0.01 | 0.21 | 0.23 |
| *vadinBE97* | 0.14a | 0.093ab | 0.079ab | 0.01 | 0.01 | 0.34 |
| *Mitochondria* | 0.10 | 0.10 | 0.09 | 0.02 | 0.53 | 0.98 |
| *Anaerolineaceae* | 0.09 | 0.11 | 0.07 | 0.02 | 0.49 | 0.19 |
| *Clostridiales_vadinBB60_group* | 0.097a | 0.074ab | 0.058bc | 0.01 | 0.02 | 0.76 |
| *Porphyromonadaceae* | 0.11 | 0.07 | 0.05 | 0.02 | 0.02 | 0.69 |
| **cecum** |  |  |  |  |  |  |
| *Ruminococcaceae* | 40.01 | 37.72 | 44.56 | 2.36 | 0.17 | 0.14 |
| *Lachnospiraceae* | 9.05 | 11.81 | 9.49 | 1.51 | 0.83 | 0.19 |
| *Prevotellaceae* | 7.24 | 7.99 | 3.91 | 1.59 | 0.14 | 0.24 |
| *Peptostreptococcaceae* | 4.94b | 4.51b | 9.34a | 0.82 | <0.01 | 0.02 |
| *Bacteroidaceae* | 7.39a | 6.88a | 3.76b | 0.88 | 0.01 | 0.25 |
| *Rikenellaceae* | 6.94 | 5.85 | 4.74 | 0.80 | 0.06 | 1.00 |
| *Christensenellaceae* | 4.20b | 4.40b | 6.58a | 0.63 | 0.01 | 0.22 |
| *Family_XIII* | 1.90 | 2.33 | 2.50 | 0.29 | 0.15 | 0.71 |
| *Acidaminococcaceae* | 1.33 | 2.79 | 0.61 | 0.96 | 0.60 | 0.14 |
| *Bacteroidales_S24-7_group* | 1.28b | 2.49a | 0.71bc | 0.36 | 0.27 | <0.01 |
| *Bacteroidales_BS11_gut_group* | 1.85 | 1.02 | 0.73 | 0.47 | 0.10 | 0.65 |
| *Peptococcaceae* | 1.13 | 1.02 | 0.89 | 0.25 | 0.49 | 0.99 |
| *Bacteroidales_Incertae_Sedis* | 1.19 | 0.77 | 0.92 | 0.32 | 0.55 | 0.49 |
| *Erysipelotrichaceae* | 0.79b | 0.79b | 1.16a | 0.10 | 0.01 | 0.14 |
| *Coriobacteriaceae* | 0.66 | 0.92 | 1.06 | 0.17 | 0.09 | 0.78 |
| *Bacteroidales_RF16_group* | 1.16 | 0.78 | 0.63 | 0.20 | 0.07 | 0.65 |
| *Clostridiales_vadinBB60_group* | 0.96 | 0.73 | 0.84 | 0.24 | 0.71 | 0.60 |
| *Porphyromonadaceae* | 0.92 | 0.86 | 0.44 | 0.15 | 0.03 | 0.34 |
| *p-2534-18B5_gut_group* | 0.88 | 0.87 | 0.46 | 0.36 | 0.41 | 0.66 |
| *Clostridiaceae_1* | 0.55b | 0.57b | 1.01a | 0.14 | 0.02 | 0.23 |

^1^ Treatments were: H_0_, basal diet without HMBi; H_15_, basal diet supplemented with 15 g/d HMBi; H_30_, basal diet supplemented with 30 g/d HMBi.

^2^ SEM = standard error of the mean.

^3^ Significant at *P* ≤ 0.05.

**Supplementary Table 3.** Effects of supplemental HMBi on bacterial composition in rumen and cecum at genus level (relative abundance > 0.5%) of beef cattle

| Genus Name | Treatment^1^ | | | SEM^2^ | *P* - value^3^ | |
| --- | --- | --- | --- | --- | --- | --- |
|  | H_0_ | H_15_ | H_30_ |  | Linear | Quadratic |
| **rumen** |  |  |  |  |  |  |
| *Prevotella_1* | 38.90 | 32.08 | 32.26 | 2.57 | 0.09 | 0.29 |
| *Christensenellaceae_R-7_group* | 6.14 | 6.50 | 6.37 | 0.43 | 0.70 | 0.65 |
| *Ruminococcaceae_NK4A214_group* | 4.83 | 5.91 | 5.03 | 0.47 | 0.76 | 0.11 |
| *Ruminococcus_2* | 3.88 | 4.75 | 4.97 | 0.62 | 0.23 | 0.68 |
| *Rikenellaceae_RC9_gut_group* | 3.46 | 3.08 | 2.98 | 0.37 | 0.38 | 0.77 |
| *Lachnospiraceae_NK3A20_group* | 1.35c | 1.93ab | 2.11a | 0.18 | 0.01 | 0.39 |
| *Succiniclasticum* | 1.37 | 1.36 | 1.85 | 0.24 | 0.18 | 0.41 |
| *Ruminococcaceae_UCG-014* | 1.59 | 2.12 | 1.95 | 0.15 | 0.12 | 0.08 |
| *Candidatus_Saccharimonas* | 1.22b | 1.67a | 1.18b | 0.12 | 0.84 | 0.01 |
| *Acetitomaculum* | 1.26 | 1.18 | 1.28 | 0.14 | 0.91 | 0.63 |
| *Saccharofermentans* | 1.06b | 1.05b | 1.37a | 0.10 | 0.04 | 0.18 |
| *Butyrivibrio_2* | 0.91 | 0.99 | 1.00 | 0.10 | 0.53 | 0.76 |
| *Prevotellaceae_NK3B31_group* | 0.33 | 0.39 | 0.48 | 0.09 | 0.25 | 0.88 |
| *Prevotellaceae_UCG-001* | 1.58 | 1.21 | 1.44 | 0.13 | 0.46 | 0.08 |
| *Prevotellaceae_UCG-003* | 1.13a | 0.92b | 0.80bc | 0.06 | <0.01 | 0.59 |
| *Lachnospiraceae_XPB1014_group* | 0.51c | 0.72ab | 0.88a | 0.07 | <0.01 | 0.75 |
| *Selenomonas_1* | 0.64bc | 0.80b | 1.72a | 0.22 | <0.01 | 0.18 |
| *Eubacterium_coprostanoligenes_group* | 0.81 | 0.87 | 0.80 | 0.06 | 0.88 | 0.44 |
| *Veillonellaceae_UCG-001* | 0.40 | 0.64 | 0.68 | 0.11 | 0.10 | 0.46 |
| *Ruminococcus_gauvreauii_group* | 0.77 | 0.65 | 0.74 | 0.08 | 0.82 | 0.29 |
| *Pseudobutyrivibrio* | 0.40bc | 0.55b | 0.80a | 0.06 | <0.01 | 0.46 |
| *Treponema_2* | 0.53 | 0.47 | 0.47 | 0.07 | 0.57 | 0.67 |
| *Ruminococcaceae_UCG-005* | 0.30 | 0.30 | 0.31 | 0.04 | 0.80 | 0.90 |
| *Ruminococcaceae_UCG-010* | 0.39 | 0.39 | 0.39 | 0.04 | 0.99 | 0.88 |
| *Anaerovibrio* | 0.30c | 0.36b | 0.61a | 0.07 | 0.01 | 0.30 |
| *Succinivibrionaceae_UCG-002* | 0.25 | 0.29 | 0.35 | 0.08 | 0.43 | 0.96 |
| *Marvinbryantia* | 0.13 | 0.19 | 0.20 | 0.03 | 0.17 | 0.58 |
| *Anaerovorax* | 0.22 | 0.24 | 0.25 | 0.04 | 0.54 | 0.96 |
| *Family_XIII_AD3011_group* | 0.25 | 0.26 | 0.28 | 0.04 | 0.59 | 1.00 |
| *Lachnobacterium* | 0.03 | 0.09 | 0.18 | 0.03 | 0.01 | 0.69 |
| *Ruminococcus_1* | 0.31b | 0.41ab | 0.61a | 0.09 | 0.02 | 0.66 |
| **cecum** |  |  |  |  |  |  |
| *Ruminococcaceae_UCG-005* | 15.81 | 15.75 | 15.30 | 0.93 | 0.69 | 0.87 |
| *Ruminococcaceae_UCG-010* | 5.82 | 5.27 | 7.00 | 1.09 | 0.44 | 0.41 |
| *Bacteroides* | 7.39a | 6.88a | 3.76b | 0.88 | 0.01 | 0.25 |
| *Eubacterium_coprostanoligenes_group* | 6.26 | 4.92 | 6.37 | 0.53 | 0.88 | 0.05 |
| *Christensenellaceae_R-7_group* | 4.12b | 4.32b | 6.43a | 0.62 | 0.01 | 0.23 |
| *Prevotellaceae_UCG-003* | 5.63 | 6.37 | 2.90 | 1.47 | 0.19 | 0.27 |
| *Rikenellaceae_RC9_gut_group* | 4.50 | 4.12 | 3.09 | 0.57 | 0.09 | 0.66 |
| *Romboutsia* | 3.12b | 2.85b | 5.56a | 0.48 | <0.01 | 0.02 |
| *Ruminococcaceae_UCG-013* | 2.34b | 2.47b | 4.26a | 0.49 | 0.01 | 0.19 |
| *Paeniclostridium* | 1.76b | 1.62b | 3.71a | 0.29 | <0.01 | 0.02 |
| *Alistipes* | 1.94a | 1.33b | 1.28b | 0.23 | 0.04 | 0.34 |
| *Lachnospiraceae_NK3A20_group* | 0.80 | 1.71 | 1.75 | 0.36 | 0.07 | 0.35 |
| *Ruminococcaceae_NK4A214_group* | 0.99bc | 1.25ab | 1.80a | 1.35 | 0.01 | 0.60 |
| *Phascolarctobacterium* | 1.28 | 1.75 | 0.57 | 0.52 | 0.33 | 0.22 |
| *Family_XIII_AD3011_group* | 0.85b | 1.30a | 1.35a | 0.15 | 0.02 | 0.29 |
| *Phocaeicola* | 1.19 | 0.77 | 0.92 | 0.32 | 0.55 | 0.49 |
| *Ruminococcaceae_UCG-002* | 1.24 | 0.47 | 0.88 | 0.86 | 0.25 | 0.05 |
| *Ruminococcaceae_UCG-009* | 0.86 | 0.77 | 0.92 | 0.11 | 0.67 | 0.36 |
| *Alloprevotella* | 0.83 | 0.93 | 0.55 | 0.27 | 0.45 | 0.49 |
| *Ruminococcaceae_UCG-014* | 0.78 | 0.50 | 0.95 | 0.16 | 0.44 | 0.08 |
| *Clostridium_sensu_stricto_1* | 0.50b | 0.50b | 1.01a | 0.67 | 0.01 | 0.10 |
| *Anaerotruncus* | 0.56 | 0.77 | 0.57 | 0.17 | 0.97 | 0.35 |
| *Tyzzerella_4* | 0.38b | 0.38b | 0.92a | 0.17 | 0.03 | 0.21 |
| *Candidatus_Saccharimonas* | 0.36 | 0.72 | 0.63 | 0.17 | 0.25 | 0.29 |
| *Turicibacter* | 0.46b | 0.42b | 0.73a | 0.08 | 0.02 | 0.08 |

^1^ Treatments were: H_0_, basal diet without HMBi; H_15_, basal diet supplemented with 15 g/d HMBi; H_30_, basal diet supplemented with 30 g/d HMBi.

^2^ SEM = standard error of the mean.

^3^ Significant at *P* ≤ 0.05.

**Supplementary Table 4.** The relative abundance of KEGG pathways of bacteria in rumen.

| Item | Treatment^1^ | | | SEM^2^ | *P* - value^3^ |
| --- | --- | --- | --- | --- | --- |
|  | H_0_ | H_15_ | H_30_ |  |  |
| Valine, leucine and isoleucine biosynthesis | 2.34 | 2.34 | 2.30 | 0.005 | 0.008 |
| Lysine biosynthesis | 1.64 | 1.64 | 1.61 | 0.005 | 0.019 |
| Cysteine and methionine metabolism | 1.30 | 1.29 | 1.27 | 0.004 | 0.015 |
| Valine, leucine and isoleucine degradation | 0.41 | 0.43 | 0.43 | 0.005 | 0.138 |
| Taurine and hypotaurine metabolism | 0.64 | 0.65 | 0.67 | 0.004 | 0.017 |
| Phosphonate and phosphinate metabolism | 0.09 | 0.08 | 0.07 | 0.003 | 0.007 |
| C5-Branched dibasic acid metabolism | 1.73 | 1.71 | 1.66 | 0.008 | 0.001 |
| Citrate cycle (TCA cycle) | 1.03 | 1.05 | 1.07 | 0.006 | 0.028 |
| Butanoate metabolism | 0.65 | 0.65 | 0.63 | 0.005 | 0.040 |
| Carbon fixation pathways in prokaryotes | 1.20 | 1.21 | 1.22 | 0.004 | 0.041 |
| Oxidative phosphorylation | 0.47 | 0.48 | 0.48 | 0.002 | 0.029 |
| Fatty acid biosynthesis | 1.55 | 1.57 | 1.51 | 0.017 | 0.225 |
| Glycerophospholipid metabolism | 0.54 | 0.54 | 0.53 | 0.002 | 0.010 |
| Linoleic acid metabolism | 0.21 | 0.20 | 0.17 | 0.007 | 0.071 |
| Folate biosynthesis | 1.06 | 1.08 | 1.13 | 0.013 | 0.099 |
| Ubiquinone and other terpenoid-quinone biosynthesis | 0.46 | 0.47 | 0.51 | 0.012 | 0.066 |
| Retinol metabolism | 0.01 | 0.01 | 0.01 | 0.000 | 0.044 |
| Lipopolysaccharide biosynthesis | 1.06 | 1.07 | 1.16 | 0.025 | 0.128 |
| Metabolism of xenobiotics by cytochrome P450 | 0.02 | 0.02 | 0.02 | 0.001 | 0.060 |
| Bacterial chemotaxis | 1.20 | 1.16 | 1.01 | 0.028 | 0.013 |
| Flagellar assembly | 0.77 | 0.75 | 0.61 | 0.025 | 0.014 |
| Phosphotransferase system (PTS) | 0.16 | 0.15 | 0.13 | 0.004 | 0.005 |
| RNA degradation | 0.59 | 0.60 | 0.60 | 0.002 | 0.107 |
| Insulin signaling pathway | 0.08 | 0.08 | 0.08 | 0.001 | 0.009 |
| Protein digestion and absorption | 0.09 | 0.09 | 0.10 | 0.003 | 0.034 |

^1^ Treatments were: H_0_, basal diet without HMBi; H_15_, basal diet supplemented with 15 g/d HMBi; H_30_, basal diet supplemented with 30 g/d HMBi.

^2^ SEM = standard error of the mean.

^3^ Significant at *P* ≤ 0.05. Only the KEGG pathways with a relative abundance above 0.1% and significant differences (p < 0.05) are presented.

**Supplementary Table 5.** The relative abundance of KEGG pathways of bacteria in cecum.

| Item | Treatment^1^ | | | SEM^2^ | *P* - value^3^ |
| --- | --- | --- | --- | --- | --- |
|  | H_0_ | H_15_ | H_30_ |  |  |
| Valine, leucine and isoleucine biosynthesis | 2.34 | 2.36 | 2.31 | 0.01 | 0.24 |
| Alanine, aspartate and glutamate metabolism | 1.75 | 1.76 | 1.72 | 0.01 | 0.01 |
| Cysteine and methionine metabolism | 1.38 | 1.40 | 1.43 | 0.01 | 0.07 |
| Histidine metabolism | 1.29 | 1.29 | 1.26 | 0.01 | 0.01 |
| Glycine, serine and threonine metabolism | 1.21 | 1.20 | 1.17 | 0.00 | <0.01 |
| Valine, leucine and isoleucine degradation | 0.43 | 0.42 | 0.40 | 0.01 | 0.05 |
| Phenylalanine metabolism | 0.27 | 0.28 | 0.26 | 0.00 | 0.03 |
| D-Alanine metabolism | 1.68 | 1.70 | 1.77 | 0.01 | <0.01 |
| Glutathione metabolism | 0.37 | 0.37 | 0.36 | 0.00 | 0.21 |
| Pentose phosphate pathway | 1.66 | 1.69 | 1.70 | 0.01 | 0.48 |
| Pyruvate metabolism | 1.23 | 1.23 | 1.27 | 0.01 | 0.05 |
| Citrate cycle (TCA cycle) | 1.09 | 1.06 | 1.02 | 0.01 | 0.03 |
| Fructose and mannose metabolism | 0.92 | 0.94 | 0.89 | 0.01 | 0.04 |
| Glyoxylate and dicarboxylate metabolism | 0.63 | 0.63 | 0.59 | 0.01 | 0.00 |
| Inositol phosphate metabolism | 0.16 | 0.17 | 0.15 | 0.00 | 0.03 |
| Oxidative phosphorylation | 0.49 | 0.49 | 0.46 | 0.00 | 0.01 |
| Glycerophospholipid metabolism | 0.53 | 0.54 | 0.56 | 0.00 | 0.04 |
| Glycerolipid metabolism | 0.43 | 0.45 | 0.47 | 0.01 | 0.09 |
| Linoleic acid metabolism | 0.38 | 0.40 | 0.36 | 0.01 | 0.03 |
| Sphingolipid metabolism | 0.33 | 0.34 | 0.25 | 0.01 | 0.04 |
| Fatty acid degradation | 0.34 | 0.33 | 0.32 | 0.00 | 0.16 |
| Pantothenate and CoA biosynthesis | 1.89 | 1.90 | 1.85 | 0.01 | <0.01 |
| One carbon pool by folate | 1.83 | 1.84 | 1.79 | 0.01 | 0.04 |
| Biotin metabolism | 1.40 | 1.43 | 1.29 | 0.03 | 0.06 |
| Vitamin B6 metabolism | 1.13 | 1.13 | 1.04 | 0.01 | <0.01 |
| Lipoic acid metabolism | 1.01 | 0.96 | 0.94 | 0.01 | 0.02 |
| Riboflavin metabolism | 0.89 | 0.90 | 0.86 | 0.01 | 0.02 |
| Porphyrin and chlorophyll metabolism | 0.74 | 0.77 | 0.79 | 0.01 | 0.07 |
| Sulfur metabolism | 0.79 | 0.80 | 0.74 | 0.01 | <0.01 |

^1^ Treatments were: H_0_, basal diet without HMBi; H_15_, basal diet supplemented with 15 g/d HMBi; H_30_, basal diet supplemented with 30 g/d HMBi.

^2^ SEM = standard error of the mean.

^3^ Significant at *P* ≤ 0.05. Only the KEGG pathways with a relative abundance above 0.1% and significant differences (p < 0.05) are presented


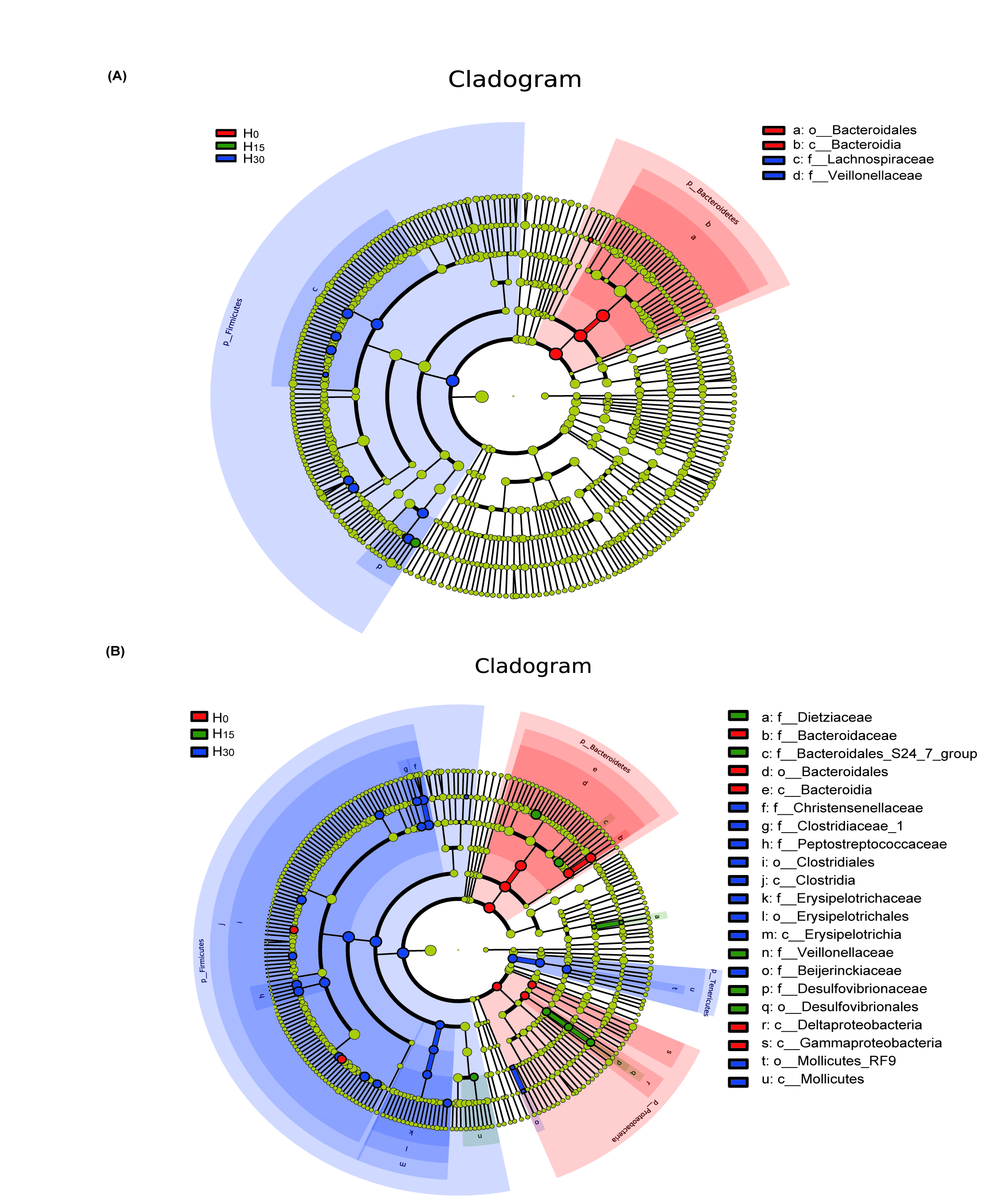


**Supplementary Figure 1.** Bacterial taxa significantly differentiated in the rumen (A) and cecum (B) identified by linear discriminant analysis effect size (LEfSe). Bacterial taxa that were differentially abundant in different groups visualized using a cladogram.
